# On-demand serum-free media formulations for human hematopoietic cell expansion using a high dimensional search algorithm

**DOI:** 10.1038/s42003-019-0296-7

**Published:** 2019-02-01

**Authors:** Michelle M. Kim, Julie Audet

**Affiliations:** 1 Institute of Biomaterials and Biomedical Engineering, University of Toronto, 164 College St, Toronto, ON M5S 3G9 Canada; 2 Department of Chemical Engineering and Applied Chemistry, University of Toronto, 200 College St, Toronto, ON M5S 3E5 Canada

## Abstract

Substitution of serum and other clinically incompatible reagents is requisite for controlling product quality in a therapeutic cell manufacturing process. However, substitution with chemically defined compounds creates a complex, large-scale optimization problem due to the large number of possible factors and dose levels, making conventional process optimization methods ineffective. We present a framework for high-dimensional optimization of serum-free formulations for the expansion of human hematopoietic cells. Our model-free approach utilizes evolutionary computing principles to drive an experiment-based feedback control platform. We validate this method by optimizing serum-free formulations for first, TF-1 cells and second, primary T-cells. For each cell type, we successfully identify a set of serum-free formulations that support cell expansions similar to the serum-containing conditions commonly used to culture these cells, by experimentally testing less than 1 × 10^−5^ % of the total search space. We also demonstrate how this iterative search process can provide insights into factor interactions that contribute to supporting cell expansion.

## Abstract (web summary)

Kim and Audet present a model-free, iterative search algorithm that optimizes serum-free formulations used to expand human hematopoietic cells. This method automatically identifies serum-free formulations that support cell expansions as effectively as the serum-containing conditions, demonstrating its utility.

## Introduction

The development of cell therapy strategies has gained traction as the interest for more personalized and novel therapeutics heightened. While the core principle of cell therapy is not new—bone marrow transplant for the treatment of leukemia is an example therapy that can trace its origins to the 1950s^1^—the main challenge of easily and efficiently obtaining compatible, safe, and competent source cells remains a challenge to this day, and is expected to pose a bottleneck in the translation of up-and-coming cell therapy strategies to the clinic. One of the common aspects that limit the efficient expansion of source cells is the requirement of serum in vitro. Serum batches vary in composition which in turn can affect the numbers and types of cell produced in culture, preventing a quality-by-design approach^2,3^.

The identification of formulations to replace serum in cell culture media^4–6^ presents a complex and difficult optimization problem as the replacement culture would require a large number of factors (cell culture supplements) in complex dose combinations. Optimizing such a large problem by conventional means such as statistical design of experiments^7^ and screening^8,9^ would be deemed infeasible due to the large number of experiments required. Alternatively, developing computational models to predict biological responses would require comprehensive mechanistic studies to identify factor effects as well as interaction characteristics. This involves many years of intense investigation, once again countering the progress and timely translation of therapies. As a result, often the only alternative is to compare among the commercially available formulations to find one that suits one’s needs.

Previous studies demonstrating drug optimization strategies relied on methods based on quadratic response surfaces of individual factors over a range of doses^10,11^ to construct models independent of mechanistic studies^12^. Recently, there has been considerable interest in combining the more conventional approach of combinatorial optimization^13,14^ with a strategy robustly used in computational and digital systems based on the Differential Evolution algorithm^15^ (Supplementary Fig. 1). The incorporation of algorithmic optimization methods (including Differential Evolution principles) have been shown to be a feasible approach for the optimization of drug combinations based on in vitro cell culture data^13,16–20^. This strategy is especially befitting in cases where discovery of combinations of multiple compounds are advantageous, but have only been applied to small scale optimization involving fewer factors (4–8 factors), requiring selective screening of multiple groups of factors, or dependent on a process that involves heavy human intervention. This approach also allows for the optimization of combinations of factors without assuming a quadratic response surface and without generating response profiles of individual factors. This is advantageous, in particular when some factors may not exhibit significant effects individually but require other factors to be present in order to act through interactions.

Herein, we present an optimization platform integrating high-throughput tools with a Differential Evolution-based algorithm that was capable of model-free navigation of a high-dimensional solution space (e.g. 15 factors at 6 dose levels) based on analyses of biological response alone. In this study, we refer to this approach as high dimensional-Differential Evolution (HD-DE). This strategy enables an automated, efficient optimization strategy for serum-free culture formulations that support cell expansion. We demonstrate the effectiveness of this approach for the identification of serum-free conditions for the expansion of two types of human cells, first in TF-1 cells (a human myeloid progenitor cell line) and subsequently in primary human T-cells for which the standard culture media used contain fetal bovine serum (FBS) and human serum, respectively. Finally, we illustrate how the data generated during the optimization process can be used to gain insights into factor potency, synergies, and dose-dependent effects.

## Results

### Development of algorithmic optimization strategy

**Fig. 1:**
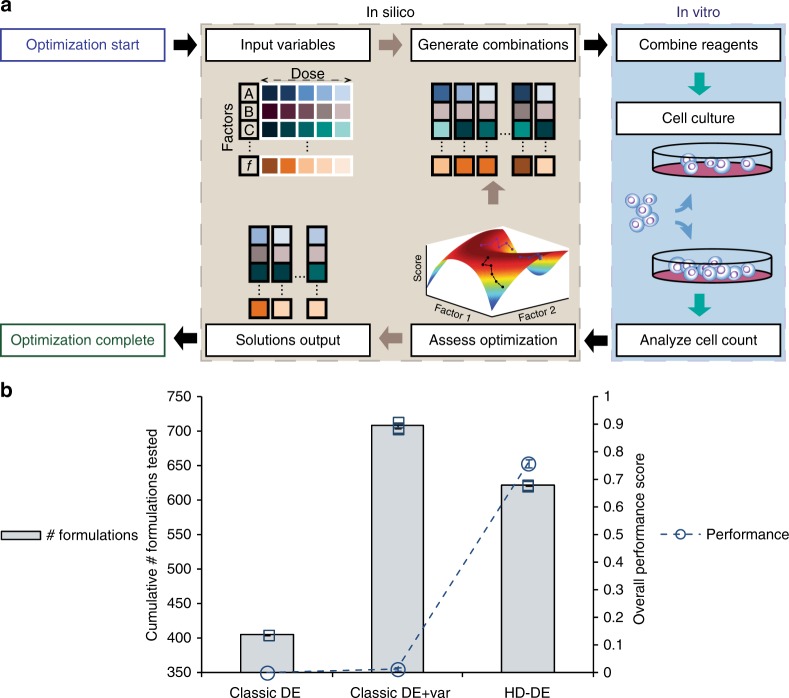
Schematic of the closed feedback optimization design leading to the improved optimization performance of the HD-DE strategy against a benchmark problem in silico. **a** All system inputs required by the algorithm (number of factors, dose levels, HD-DE parameters, decision-making criteria) were pre-defined. The algorithm-generated test formulations were compounded according to the corresponding recipe and cells cultured. The final cell count (system response) was input into the algorithm which analyzed the response and iterated (further optimized) or terminated. A single cycle from generating combinations through in vitro culture, analysis, evaluation, and decision to iterate or terminate made up a single generation (abbreviated to “*G*(*n*)” with *n* = number of generation) of the HD-DE-driven optimization process. For in silico simulations, a benchmark function and a normal random variable was used to generate the response in lieu of cell culture. The result of evaluating the benchmark function for each given formulation was treated as the system input equivalent to the biological response. **b** The classical Differential Evolution was ineffective in improving the overall performance against a high-dimensional benchmark problem such as the Rosenbrock function. Recognition of the variability (“Classic DE + var”) in the simulated data produced some improvement in the overall performance at the expense of more formulations being tested. Additionally, the introduction and utilization of information collated in the self-assembled data library (HD-DE strategy) enabled selective exploration and clearing of candidate formulations, further improving performance and efficiency of the overall optimization process. Data presented three-independent sets for Classic DE and Classic DE + var conditions, and eight-independent sets for HD-DE condition. Data represent mean ± standard error of the mean (SEM)

Based on a number of previous studies^16–18^ supporting the capability and resilience of the Differential Evolution algorithm in the optimization of cell system conditions, the performance of the Differential Evolution algorithm was assessed on a larger, more complex optimization problem than demonstrated in any previous studies. Modifications required to the classic Differential Evolution algorithm were designed to improve efficiency and to accommodate the challenges in optimizing complex cell culture systems. An experiment-based feedback control platform enclosed all system inputs, parameters, and decision-making parameters in a self-contained system for the optimization to run independent of introduction of prior knowledge regarding downstream mechanisms, interactions, models, and selection bias (Fig. 1a). This closed feedback control strategy involved a phenotype-driven optimization approach which based its decisions on the overall response of the system to identify an improved state. The response of interest in this study was the degree of cell expansion, and the analyses of this experimental data from the various formulations tested was used to navigate the solution space. The variability present in the system was recognized by introducing a statistical component in the design of the optimization platform based on the variance observed in the experimental results. Benefits of such approach to better represent the range of biological responses^21^ have also been demonstrated in our prior computational modeling studies where cell system-level experimental variability was incorporated in in silico models. This addition of variability consideration resulted in the smoothing of the overall landscape reducing the prominence of local maxima. A self-assembled library of test formulations and results provided a robust and reliable foundation for identifying potential solution regions and providing directional guidance to pursue selective exploration and clearing^22^, accompanied by a responsive optimization strategy based on feedback of experimental results (Supplementary Fig. 2). The initial focus on exploration of the solution space and concurrent pursuit of multiple optimization routes maintains a test population size through the process and further encourages the discovery of regions of improved performance. This responsiveness of the HD-DE approach conserved the goal-driven motivations of conventional adaptive strategies using simpler methods compared to strategies which combined multiple mutation and crossover operations^23^. For comparison, a performance score was calculated, which was the cell expansion obtained with the encountered test formulations normalized to the maximum cell expansion achieved by the serum-containing “Positive Control” (PC) i.e. cell expansion achieved using commonly used serum-containing culture formulation for a given cell type.

Prior to experimental optimization in vitro, several versions of the algorithm were evaluated in silico by executing a test optimization process using the Rosenbrock function^24^ as the benchmark^25,26^ to test a 15-dimensional optimization problem (i.e. 15-factors, each spanning 5 dose levels). A normal random variable was incorporated into the objective function in order to provide a more realistic simulation of the acquisition of a biological response (i.e. an experimental result with variance) to each formulation. The normalized response score (i.e. normalized to the known maximum of the function or PC) and the number of candidate solutions were identified as the critical indicators that measured the algorithm performance by reflecting the successful convergence of the optimization solutions candidate solution set (Fig. 1b, Supplementary Data 1). This set-up established a performance evaluation structure that was maintained throughout in vitro data acquisition. As expected, the performance of HD-DE was superior than a random selection of a similar or greater total number of formulations scored on the same benchmark as the in silico runs (see Supplementary Discussion).

### Robustness of optimization capacity

Next, the HD-DE algorithm previously tested in silico was given an in vitro high dimension optimization problem of 15 cell culture factors comprising of supplements such as small molecule inhibitors, growth factors, and nutrient compounds, each at up to 6 doses (Supplementary Table 1). These experiments were aimed at optimizing the serum-free culture of TF-1 cells, a cytokine-dependent human hematopoietic progenitor cell line^27^. The factors were selected with the intent to replace the fetal bovine serum (FBS) added to the cell culture medium and to reduce the concentration of granulocyte-macrophage colony-stimulating factor (GM-CSF) required. The iterative feedback control (Fig. 1a) was implemented to generate test formulations (factor-dose combinations) and analyze responses with the aim of promoting cell expansion of TF-1 cells in the absence of serum. The profile of algorithm performance and behavior from in silico optimization against a benchmark function with a known solution (Supplementary Fig. 3) was used as a baseline to monitor the progress of the in vitro optimization using HD-DE. The in vitro validation of the HD-DE optimization was performed by comparing the cell expansion achieved by the serum-free formulations to that of the serum-supplemented positive control (PC) condition. While not a direct assessment of optimization performance within the defined parameters (Supplementary Table 1), this provided a performance standard to which the HD-DE could be compared.

**Fig. 2:**
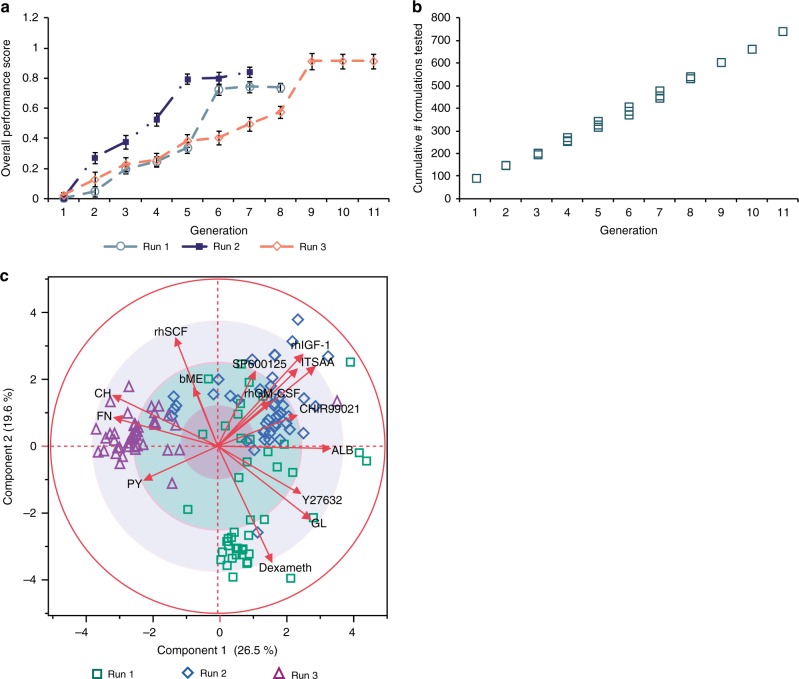
In vitro results from three-independent experimental runs demonstrate robustness of the HD-DE optimization process for TF-1 cells. **a** The algorithm was able to identify optimized conditions that sustained cell expansion under serum-free formulations. Variation in overall performance underscored the presence of variability in biological systems. The performance was normalized to that of the known maximum score (PC of TF-1 cell culture). **b** Despite variability, the algorithm was able to keep the optimization cost under control as it utilized the information gathered. **c** PCA loading illustrated the degree to which factor doses were conserved in the candidate solution set formulations. (See Supplementary Table 1 for factor legend). Data represent mean ± SEM

To test the robustness of the HD-DE algorithm-guided optimization process, we repeated the search for serum-free formulations in three-independent experiments, in run 1–3, which were biological replicates initiated from three different cryopreserved TF-1 cell batches. Each run started with testing of a unique set of formulations (initial conditions). Figure 2a indicates that each trial followed a distinct path to optimization. However, a similar level of cell expansion was reached at convergence, demonstrating the ability of the HD-DE approach to deal with different initial conditions and the variability inherent to biological experiments. The in vitro optimization process was handled by the algorithm in a similar manner as in silico with the number of formulations with improved cell expansion between consecutive generations decreasing and formulations with no further improvement, increasing at later generations (Supplementary Fig. 3, Supplementary Fig. 4a, b). Despite the large solution space presented to the algorithm (Supplementary Table 1), identification of serum-free formulations resulting in levels of cell expansion comparable to the serum-containing PC condition was achieved with the testing of less than 800 unique formulations (Fig. 2b).

Further evaluation using principal component analysis (PCA) of the formulations of the final candidate solution set suggested that the algorithm identified similarly performing formulations (Fig. 2c) clustering in various regions of the solution space from distinct initial conditions. Additionally, while the solution regions may differ, the overall clustering characteristics and relationship among the formulations of the candidate solution set as measured by Hamming and Levenshtein distances (Supplementary Fig. 4c, d) remained consistent. Based on these observations, the relative composition of the candidate solution set formulations was further analyzed. The loading of each factor from PCA (Fig. 2c, red arrows radiating outwards from origin) showed the varying degree to which each factor was conserved among the final candidate solution set formulations. Conventionally, factors with significant effect on the overall response were represented to be further away from the origin (0, 0) of the PCA biplot. As the formulations consisted of final solutions, the effect of any single factor on the overall response bore less significance. Considering only the distance from the origin, the more conserved a factor dose was among the candidate solution set formulations, the PCA loading determined the factor to have less effect on the overall response, positioning the factor closer to origin. This applied regardless of the nature of the factor effects, which also correlated to the estimated factor effects elucidated from other analyses (Supplementary Fig. 5, Supplementary Fig. 6). The pattern of conserved factors and doses was also reflected in the composition breakdown of the candidate solution sets for each experiment, and in the comparison between experiments (Supplementary Fig. 6, right panels). Positive-effect factors consistently had greater proportion of formulations with high-dose level of such factors, while negative effect factors had greater proportion of formulations with these factors at low dose levels.

### Optimization of human T-cell expansion culture formulations

**Fig. 3:**
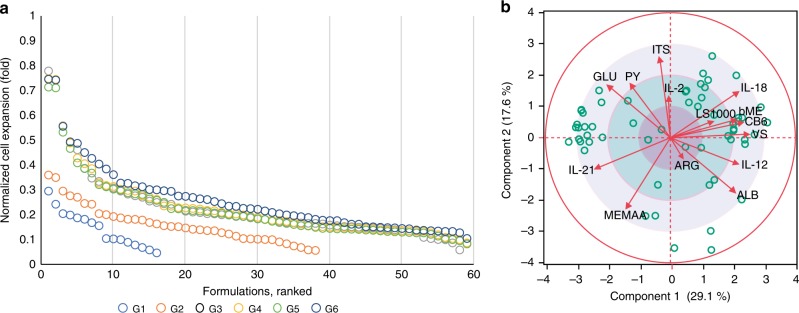
In vitro media optimization performance of 14-factor T-cell culture demonstrated applicability of HD-DE strategy to the optimization of primary cell culture formulations. **a** Ranking of individual formulations of the candidate solution set according to the score expressed as the cell expansion normalized to that of the PC condition for T-cells showing change through generations. A number of formulations that supported T-cell expansion at levels comparable to that of the PC condition were identified. **b** PCA biplot illustrate the distribution of identified formulations in the solution space and indicate the degree to which factors are conserved among the identified formulations. (See Supplementary Table 2 for factor legend)

Following the in vitro optimization of serum-free media of TF-1 cells, we used the same optimization strategy to identify and optimize serum-free media formulations for primary human T-cell culture. 14 factors (Supplementary Table 2) and their corresponding dose levels were defined, and the optimization process was executed using frozen cell aliquots from a single donor, and performance was compared to the positive control (PC) condition for T cells (a serum-supplemented formulation for T-cell culture). By the end of 6 generations, the optimization process was able to identify a number of formulations that supported comparable levels of cell expansion (i.e. 70–80%) to the PC (Fig. 3a). Less than 600 unique formulations were tested in the 6 generations (Supplementary Fig. 7a, b), following a trend similar to that observed in the previous optimization using TF-1 cells. The overall performance of the candidate solution set at each generation followed the expected behavior where the number of formulations that were further improved between consecutive generations decreased at later generations. Concurrently, the number of formulations carried over between consecutive generations increased and converged towards the population size (Supplementary Fig. 7c, d). This resulted in an increase in the overall similarity at later generations (Supplementary Fig. 7e). While the overall behavior of the optimization was consistent with previous observations with TF-1 cells, fewer formulations achieved the same level of performance score as previously observed for TF-1 cells were identified (Supplementary Fig. 7a); the final measure of similarity of the candidate solution set formulations also reflects this result with a mid-density cluster exhibiting high similarity (low Hamming Dist. and low Levenshtein Dist.), and the majority of the formulations clustered further away (Supplementary Fig. 7f). The composition of the formulations of the final candidate solution set analyzed by PCA provided an indication of the factors and dose levels most conserved among the final formulations. The PCA biplot revealed that arginine (ARG) was the most highly conserved factor (Fig. 3b) at the mid-to-high-dose levels (Supplementary Fig. 7g).

### Robustness across cultures originating from different donors

**Fig. 4:**
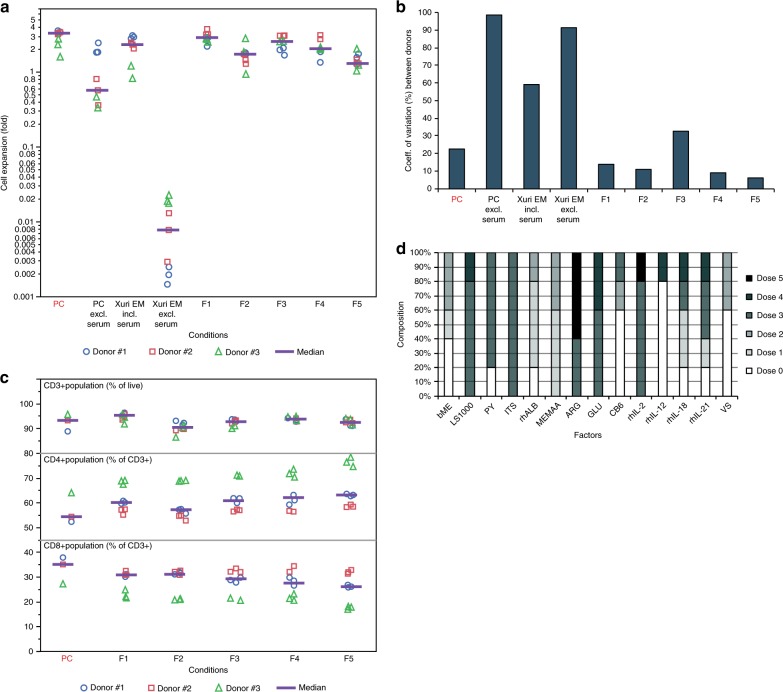
Characterization and comparison of the expanded T-cell population cultured using the top 5 serum-free formulations identified. **a** Comparison of the variation in cell expansion between donors for the variation of the media conditions. **b** Comparison of cell expansion capacity between two commercially available serum-free media formulations (PC and Xuri EM) with and without serum supplementation and the top 5 formulations identified (F1–F5). Cell expansion was compared across the three donor cells. *p*-values using ANOVA and post hoc Tukey’s multiple comparison tests listed in Supplementary Data 2. **c** The top 5 formulations analyzed for cell populations expressing T-cell markers CD3, CD4, and CD8. *p*-values using ANOVA and post hoc Tukey’s multiple comparison tests listed in Supplementary Table 3. **d** Analysis of the composition of the top 5 formulations (Supplementary Table 4) identified

The top five formulations in T-cell cultures were selected, and the cell expansion capacity of these formulations was compared to those obtained from cultures using commercially available formulations. The two commercial formulations (XVIVO-15 already used as base media for PC and XuriEM) were tested under conditions including and excluding serum supplementation. The serum-free formulations identified through the HD-DE optimization (F1–F5) supported cell expansion at levels comparable to those observed under serum-containing conditions (Fig. 4a, Supplementary Data 2). Upon analysis of the variation in cell expansion that occurred between the donor cells, the relative variability observed from culture using F1–F5 formulations were comparable to or less than that observed for cells cultured in serum-supplemented media conditions and less than half the variability level observed in commercial media used without serum supplementation (Fig. 4b). The resulting expanded cell population from cultures using the 5 serum-free formulations was further characterized by identifying a set of cell surface markers specific for T-cells. The overall composition of the population of CD3-expressing cells displayed to be consistent across markers and formulations at levels comparable to those observed in the cells of the serum-containing expansion culture (PC condition) (Fig. 4c, Supplementary Table 3). The composition of the top 5 formulations (Fig. 4d, Supplementary Table 4) showed an amplification of the most prevalent dose levels (Supplementary Fig. 7g) for many of the factors suggested to be highly conserved among the candidate solution set formulations (Fig. 3b).

### Insights from post hoc multivariable analysis

**Fig. 5:**
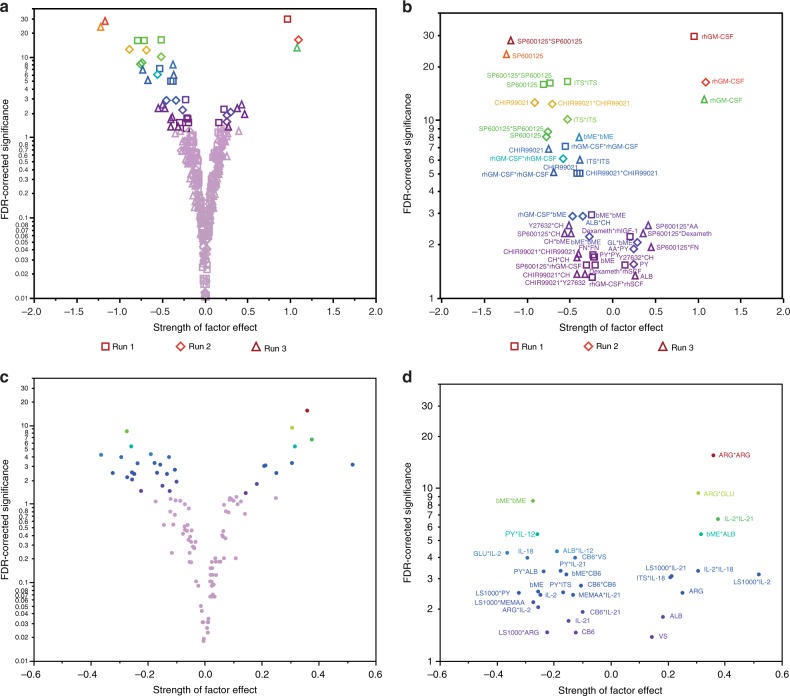
Multivariable analysis of the dataset obtained through HD-DE optimization in vitro. **a** The full test library of formulations obtained from all 3 runs of the HD-DE optimization performed on TF-1 cells (excluding *G*(1)) was used for post hoc analysis to elucidate the main effects, quadratic effects, and 2-factor interactions. The logworth statistical significance of the regression coefficient estimates are presented in a plot where the significant effects (false discovery rate (FDR)-corrected *p*-values <0.05) are colored while non-significant elements are grayed. **b** The data points that have significant effects (colored data points from (**a**)) are labeled with the corresponding factor or interaction. **c** The full test library of formulations obtained from the HD-DE optimization performed on T-cells (excluding *G*(1)) was used for post hoc analysis to elucidate the main effects, quadratic effects, and 2-factor interactions. The FDR-statistically significant effects (expressed as logworth *p*-values) are colored while non-significant elements are grayed. **d** The data points that have statistically significant effects (colored data points from (**c**)) are labeled with the corresponding factor or interaction. The square term suggest either a supra-additive relationship (positive term) between effect and dose or a saturation (negative term), and the cross-product terms refer to 2-factor interactions. (See Supplementary Table 2 for factor legend)

The phenotype-driven optimization process of the culture conditions generated its own knowledge library consisting of the composition of tested formulations and their corresponding biological response. Once the search for optimal factor combinations was completed, this library was used to conduct a post hoc multivariable analysis to elucidate the main factors and possible interactions that significantly contributed to the overall response versus those that were less important. The data were fitted to a polynomial quadratic model (Equation (1)) where the regressors correspond to factor main effects (i.e. linear dose effects), two-factor interaction effects (synergies or antagonisms), and quadratic effects (non-linear dose effects)^28^. For this analysis, the amplitude, direction, and statistical significance of the regressors were calculated (see Supplementary Data 3). The results of these analyses are presented in volcano plots (Fig. 5a, b for TF-1; Fig. 5c, d for T cells) where the negligible factor effects tend to fall near zero on the horizontal axis (effect strength) and low on the vertical axis (FDR logworth statistical significance). On the other hand, the most important positive effects supporting cell expansion would be found on the top right quadrant of the volcano plot and the dominant-negative effects, on the top left. This analysis was performed on each of the three replicated experiments (runs) with TF-1 cells (Fig. 5a, b). As expected, rhGM-CSF was consistently the most potent factor for TF-1 cell expansion (Fig. 5b, Supplementary Table 5), with evidence of high-dose saturation (negative quadratic effect). For T cells, ARG had the strongest-positive effect, in particular at the highest doses of the range covered (no saturation), as indicated by its significant positive quadratic effect. SP600125 and CHIR99021 consistently had a pronounced negative effect on TF-1 cell expansion, although some of the negative effects were attenuated to some extent by the presence of other factors (as revealed by the number of significant positive two-factor interactions in which either the small molecules SP600125 or CHIR99021 were involved). Some other negative effects seemed to occur mainly at high doses (ITS and bME for TF-1 in Fig. 5b; bME and CB6 for T-cells in Fig. 5d), as suggested by the predominance of the negative quadratic effects relative to the main effects. Both TF-1 and T-cell expansion seemed to be controlled by a large number of weaker positive and negative two-factor interactions and a small number of dominant main factor effects. As expected, the detection of the strongest and most significant effects tend to be consistent across the replicates of TF-1 experiments. On the other hand, the weaker (but still significant) effects were not detected in all of the TF-1 experiments. In this analysis, the two-factor interactions that are in the upper right section of the volcano plot are suggestive of factor synergies (with the condition that the main and quadratic effect component of these two factors are not significantly negative). While strong factor synergies were not detected in the case of TF-1 cells, there was at least one in T-cell culture such as the synergy between ARG and GLU (ARG*GLU in Fig. 5d). Interestingly, among the cytokines tested in T-cell culture (i.e. IL-2, IL-12, IL-18, and IL-21) none had a significant positive main effect while most had a significant positive interactions with at least one of the other cytokines (e.g. IL-2*IL-21 in Fig. 5d). This suggests that these cytokines cannot act on their own to promote T-cell expansion and must signal in concert with others.

It is also possible with this analysis to find evidence of factor(s) that may be inert. Inert factors would be expected to have no significant main or quadratic effects and not be part of any significant interactions. We found that rhIGF-1 and GL met these criteria but only in some of the TF-1 experiment replicates (note that this is not unexpected for GL given that it was already present in the base culture media for TF-1 cells). We did not find strong evidence of inert factors in T-cell culture, but ITS, IL-12, and MEMAA were the least active factors among the ones tested. The quadratic model used for the post hoc analysis is a simple approximation and, therefore, is more accurate to study response surfaces that are smoother^10,12^ than those with a more rugged topography. Another limitation of the post hoc analysis is that only two-factor interactions were taken into account in the model and higher-order interactions (i.e. beyond two factors) were assumed to be negligible^29^. Nonetheless, the most important factors that were identified for TF-1 and T-cell expansion by multivariable analysis (Fig. 5, Supplementary Data 3 and Supplementary Fig. 8) agreed with the results of similarity analysis (Supplementary Fig. 4c) and qualitative observations (Supplementary Fig. 5).

## Discussion

The proof-of-concept study presented here demonstrated the ability of HD-DE, a closed feedback optimization system to identify serum-free culture formulations for human hematopoietic cells from a large, complex solution space. The optimization process was guided by the HD-DE strategy, where the improvement in overall efficiency was aided by selectively navigating towards candidate solution regions based on those identified in the early generations, overcoming limitations of conventional optimization methods such as restrictions on the number of factors and doses that can be considered in combination^30^. As demonstrated by the correlation observed between the similarity and multivariable analyses, further information regarding the mechanisms governing the composition of the formulations that promote cell expansion can be derived upon completion of the optimization. For instance, a post hoc multivariable analysis of the serum-free formulations identified through HD-DE optimization generated specific hypotheses regarding factor synergies and also “silent” factors. This was made possible by pointing to specific factor effects among a list of over a hundred other candidates (including all main, quadratic and two-factor interaction effects). This is a considerable benefit since it now becomes possible to design follow-up experiments to elucidate the particular cellular mechanisms at play. Therefore, further assessment of the formulations that were identified by HD-DE could provide a starting point to further optimize, or reduce, the culture compositions in a time- and cost-effective manner.

The HD-DE optimization strategy was applied to the discovery of serum-free media formulations for human primary T-cell expansion culture, demonstrating the applicability and feasibility of executing a complex optimization process in a model-free, phenotype-based algorithm-guided environment integrated with high-throughput automation. The number of individual formulations with T-cell expansion capacity comparable to the PC condition was smaller than in the case of TF-1 cell culture. However, the serum-free formulations identified produced cell expansion populations with characteristics comparable to those expanded in conventional serum-containing cultures (PC). More importantly, in some of the serum-free formulations discovered, T-cell expansion was an order of magnitude higher than those expanded in cultures using commercially available serum-free media formulations. It is important to note that the total costs of reagents for the formulations identified were similar or lower than commercially available serum-free media.

Interestingly, the top-performing serum-free formulations identified through the HD-DE strategy seemed to display lower donor-to-donor variability in T-cell expansion compared to the commercially available formulations used without serum supplementation. This can be advantageous as the use of robust formulations free of animal- or human-derived serum supplementation can be used earlier in the development process and easily modified to suit various cell types and needs. The use of serum-free formulations would also ease the standardization of reagent quality and reduce the complexity of the regulatory process expediting the translation and delivery of cell therapy products.

The HD-DE optimization platform presented in the current study demonstrated the feasibility of adopting an algorithm-guided, high-dimensional optimization approach in the identification and optimization of formulations for T-cell expansion culture. The identified serum-free formulations can be further developed into processes for applications, such as CAR T-cell therapy. Treating the biological system as a “black box” may also be particularly useful for a cell type that has yet to be fully characterized, or applied in other optimization situations that deal with complex problems involving a large number of factors such as in cryopreservation protocols to improve cell recovery upon thawing^16^. The success of the optimization process is dependent on the definition of the problem consisting of the types of factors as well as the dose range, which are parameters defined prior to start of the optimization process and ones that can be established without the need for extensive mechanistic studies. The overall optimization approach of phenotype-based decision-making suggests the possibility of establishing a standardized, universal optimization protocol for any cell type or requirement, making it possible to obtain culture medium with specific characteristics on-demand which will dramatically facilitate the implantation of a quality-by-design approach. This optimization strategy also presents a blueprint to fully automate the optimization process in a high-throughput manner.

## Methods

### Compounding culture factor combinations for TF-1 cells

The epMotion 5070 liquid hander (Eppendorf) was used to compound the culture condition “recipe” according to the prescribed algorithm-generated test formulations. Each factor was diluted to the appropriate concentration in a base media of DMEM (Gibco #12430054) supplemented with 1% Penicillin-Streptomycin (Pen-Strep; Gibco #15140122) and distributed into 48-well plates. The 15 factors selected to supplement the base media were Glycogen synthase kinase inhibitor (CHIR99021)^19,31^, Jun N-terminal kinase inhibitor (SP600125)^32^, dexamethasone (Dexameth)^4^, granulocyte macrophage-colony-stimulating factor (rhGM-CSF), stem cell factor (rhSCF)^33^, insulin-like growth factor 1 (rhIGF-1)^4^, ascorbic acid (AA)^4^, Rho kinase inhibitor (Y27632)^19^, albumin (ALB)^33^, fibronectin (FN)^4^, GlutaMAX™ Supplement (GL)^4,5^, cholesterol concentrate (CH)^4^, ITS Supplement (ITS)^4,33^, β-mercaptoethanol (bME)^4,33^, and sodium pyruvate (PY). The dose ranges tested for each factor are listed in Supplementary Table 1.

### TF-1 cell maintenance

TF-1 cells (ATCC #CRL-2003) were maintained in recommended complete growth medium (RPMI 1640 (Gibco #22400089) supplemented with 10% FBS (Gibco #12483020), 2 ng per ml recombinant human Granulocyte-macrophage colony-stimulating factor (rhGM-CSF; R&D Systems #215-GM-010), and 1% Pen-Strep in T25 or T75 flasks at 37 °C with 5% CO_2_.

### T-cells preparation

T-cells were isolated from peripheral blood mononuclear cell (PBMC). Buffy coats (Canadian Blood Services; Donor #2, donation # C0510172128482; Donor #3, donation # C05101721879820) were diluted with equal parts volume of sterile buffer of Dulbecco’s phosphate-buffered saline (DPBS, GE Healthcare) with 2% Human Serum AB (Gemini Bio-products). PBMCs were isolated using Ficoll-paque PLUS (GE Healthcare) according to manufacturer guidelines. The cells were washed by centrifugation once again, and resuspended in 10 ml of complete growth media (XVIVO CGM) consisting of XVIVO-15 (Lonza) supplemented with 1% PenStrep, 5% human serum, 1% SG-200 (GE Healthcare), and 350 IU per ml recombinant human Interleukin 2 (IL-2, GE Healthcare). The cells prepared in the individual tubes were combined into a single tube and XVIVO CGM added to a final volume of 80 ml.

CD-positive (CD3+) T-cells were isolated from PBMCs, either from Buffy Coats or LeukoPak apheresis units (Stem Cell Technologies; Donor #1), as a CD3+ depletion fraction of a CD3-CD56+ NK-cell isolation process. The sequential separation of cell populations utilized positive selection of the CD3+ fraction using magnetic microbeads and the CliniMACS® System or MACS® Columns (Miltenyi Biotec) according to manufacturer instructions. The isolated T cells were resuspended in CryoStor-10 (CS10, BioLife Solutions) cryopreservation media at aliquot sizes of 20 × 10^6^ or 40 × 10^6^ cells.

Canadian Blood Services approved REB application for work using donor blood material conducted at CCRM, an approved CL2 facility with appropriate guidance and procedures complying with all relevant ethical regulations, for research purposes.

### Compounding culture factor combinations for T-cells

The factors were compounded using the Nimbus Microlab Liquid Handling System (Hamilton Robotics) in basal media of DMEM/F12 + 1% PenStrep. Cryopreserved CD3+ cells from DN1 were thawed in 10 ml of basal media supplemented with 7% Bovine Serum Albumin (Sigma; stock solution prepared to 200 mg per ml in DPBS). The cells were centrifuged (all centrifugation steps hereon at 400 g for 10 min unless otherwise specified) and the pellet washed and resuspended in plating media (basal media supplemented with 2% Human Serum Albumin (Sigma; stock solution prepared to 200 mg per ml in DPBS)). The cells were then counted and resuspended at target density for seeding in plating media and activated with the addition of CD3/CD28/CD2 T-cell activator (Stem Cell Technologies) according to manufacturer dosage instructions. The 14 factors selected to supplement the base media were β-mercaptoethanol (bME)^34–36^, LS1000 Lipid Supplement (LS1000)^37^, sodium pyruvate (PY)^34,38^, Insulin-Transferrin-Selenium-Ethanolamine (ITS -X)^35,39^, albumin (rhALB)^35,37,40,41^, MEM non-essential amino acids solution (MEMAA)^37,39^, L-arginine (ARG)^38^, SG-200 solution (GLU)^38,42^, Cell Boost™ 6 (CN-T) supplement (CB6), IL-2 growth factor (rhIL-2)^34,38,43^, Interleukin 12 (rhIL-12)^34,38^, Interleukin 18 (rhIL-18)^34^, Interleukin 21 (rhIL-21)^44^, and MEM vitamin solution (VS)^37^. The dose ranges tested for each factor are listed in Supplementary Table 2.

### TF-1 test combination culture

Upon completion of the compounding of culture factor cocktails using the liquid handler, the cells were washed three times and resuspended in DMEM+1% Pen-Strep. The cell suspension was allocated to each well at a seeding density of 30,000 cells per ml and a total culture volume of 500 μl per well which were incubated for 5 days. The serum-containing culture condition (usual supplements added to a base medium of DMEM instead of RPMI 1640) was used as the “Positive Control” (PC) condition.

### TF-1 live cell count

The cell suspension was dissociated using TrypLE (Gibco), transferred into 96-well V-bottom plates, washed with PBS, and resuspended in HBSS+2% FBS with 1:1000 7-Aminoactinomycin D (7-AAD; Molecular Probes). The numbers of live cells in each well were counted using the HTS platform on the BD LSRFortessa flow cytometer (BD Biosciences) (Supplementary Fig. 9).

### Live cell count and T-cell phenotype characterization

On day 5, the culture plates were centrifuged and washed with DPBS to remove remnants of the culture media. The cells were incubated for 10 min with 30 μl TrypLE (Thermo Fisher) without fully dissociating the aggregates. All test wells with the exception of the unstained sample were resuspended with 70 μl Flow buffer (DPBS+2% HS+1 mM Ethylenediaminetetraacetic acid solution (EDTA, Sigma)) including 1:1000 7-AAD. The aggregates were fully dissociated by gentle pipetting just prior to initiation of the count protocol where the number of viable cells was counted using the CytoFlex (Beckman Coulter) in plate mode, sampling at 90 μl per min for 40 s.

The selected formulations and cells were prepared as illustrated in previous sections. On day 5, the culture plates were centrifuged and washed with DPBS to remove remnants of the culture media. The cells were incubated for 30 min at 4 °C in the dark with 50 μl T-cell flow cytometry panel master mix. After the incubation period, 100 μl DPBS was added to each well and the plates centrifuged. The cells were resuspended in 100 μl Flow buffer and the aggregates fully dissociated by gentle pipetting just prior to initiation of the count protocol where the number of viable cells were counted using the CytoFlex (Beckman Coulter) in plate mode, sampling at 90 μl per min for 40 s (Supplementary Fig. 10).

### Coding and statistical analyses

The algorithm was written in MATLAB (Mathworks) and executed on a Windows 8.1 device. The initial conditions of the native Differential Evolution parameters governing mutation (*F*) and crossover (CR) (Supplementary Fig. 1) were defined as *F* = 1 and CR = 0.5. The values of these parameters were changed according to the progression of the optimization, where *F* was reduced to 0.5 upon detection of convergence of the overall score. Following the reduction in *F*, CR was reduced to 0.25 when at least half of the elements in the target formulation (*X*_*i*_ in Supplementary Fig. 1) were not changed between two consecutive generations. For the in silico and in vitro TF-1 cell media formulation optimization runs, a population size of 45 generated by 3 × *D* (where *D* corresponded to the number of factors, 15) was used. For the in vitro T-cell media formulation run, a population size of 59 (3 × *D* + 17 extra test formulations to utilize the increased capacity of the liquid handling platform) was used. The in silico validation of the algorithm, including generation of simulated response data points, lasted for ~40 min for each run. The comparison of HD-DE with random selection (Supplementary Fig. 11) used the same benchmark as the in silico runs. For the in vitro experiments, the optimization parameters were defined within MATLAB and the algorithm was used to generate the test combinations. The reagent transfer commands and subsequent results of the in vitro culture were directly transferred from and from the MATLAB environment to the liquid handler interface and from the flow cytometer software into MATLAB in the form of CSV files. The post hoc multivariable analysis and principal component analysis (PCA) were performed using JMP12 (SAS) on the same Windows 8.1 device. For the multivariable analysis, all tested formulations excluding the initial populations from all 3 TF-1 cell experimental runs were combined to generate the dataset. For T-cells, only one dataset was available. Using JMP12, the response was then log-transformed. Imputation of left-censored data was performed by estimating a normal distribution of the response below count sensitivity threshold. A quadratic polynomial model was fitted by least square regression using the response screening platform in JMP. The equation included all quadratic (square) terms and all two-factor interactions (crossproduct) terms in equation (1):

1 $$Y = K + Gp + \mathop {\sum }\limits_{j = 1}^D \beta _jx_j + \mathop {\sum }\limits_{i = 1}^D \mathop {\sum }\limits_{j = 1}^D \beta _{ij}x_ix_j + \mathop {\sum }\limits_{j = 1}^D \beta _{jj}x_j^2 + {\it{\epsilon }},$$

where *Y* corresponded to the log-transformed values of cell expansion, *K* corresponded to the intercept, *G*_*p*_ was the block parameter for the *p*^th^ generation, *D* corresponded to the number of factors, *β*_*j*_ corresponded to the main effect coefficient for factor *j*, *β*_*ij*_ corresponded to the interaction effect coefficient between factors *i* and *j*, *x*_*j*_ corresponded to the coded dose [−1, 1] for factor *j*, *x*_*i*_ corresponded to the coded dose [−1,1] for factor *i*, and *ε* corresponded to the random error (residuals). The statistical significance of the regression coefficient estimates were false discovery rate (FDR)-corrected^45^ for *p*-values <0.05. The values for the *β*_*i*_, *β*_*ij*_, *β*_*jj*_ and corresponding FDR *p*-values are provided for each experiment in Supplementary Data 3. PCA was conducted on the compiled dataset of formulations of the final candidate solution set from all 3 experimental runs.

### Algorithm analysis and selection

Multiple competition rounds between formulations within and across generations was incorporated for formulation selection and induction into the candidate solution set. All analyses were completed by the algorithm upon input of test response (viable cell number count), generating either output of test formulations for the next generation or determining the termination or completion of the optimization process. The competition required was composed of three main elements. The first element, is competition within test generation where the target formulations versus trial formulations within each generation were compared according to the Wilcoxon rank-sum test. The winning individual member of the population advanced to next round. The second element is competition against best encountered where the formulations previously selected in the candidate solution set were compared with any new formulations identified with consideration given to the estimated inter-experimental variability to select the better of the two. The third element was clearing/niching where, at later generations, formulations that scored outside of a pre-defined threshold of a 10% score range of candidate solution set formulations were actively replaced out by a clearing mechanism. A combination of stochastic selection and deterministic perturbation of the composition of root formulations was used to generate a pool of candidate formulations from which a test formulation was randomly selected.

### Similarity analysis

Two metrics analyzing similarity were adapted to assess the degree of similarity between two formulations at consecutive generations. First, the Hamming distance^46^ counted as the number of factors in a query formulation with dose level designation not equal to the dose of the same factor in the reference formulation. Second, the Levenshtein distance^47^ measured as the sum of dose levels difference between the query and reference formulations overall factors. Formally, the Levenshtein distance counts the number of edits (substitution, insertion, or deletion of an element) required to change one formulation sequence to the other. As the formulations being compared are of equal length, counting insertion, or deletion edits become meaningless. Unlike comparison of two text sequences, where such similarity measurements are often used, each element of the formulation can vary across a range of doses coded and represented as 0, 1, 2, 3, … This aspect of dose levels for each element was introduced to the substitution count of the Levenshtein distance measurement by considering the discrepancy in the number of doses between the corresponding elements in the query and reference formulations. For the purposes of formulation analysis, the Levenshtein-equivalent distance (hereon referred to as ‘Levenshtein distance’) measured the total number of dose level discrepancies across all elements of the formulation sequence.

### Code availability

The custom code is available at GitHub (https://github.com/julieaudet/cell-manufacturing).

### Reporting summary

Further information on experimental design is available in the Nature Research Reporting Summary linked to this article.

## Supplementary information

Supplementary Information

Reporting Summary

## Data Availability

The datasets generated during and/or analysed during the current study are available in figshare^48^, which include Supplementary Data 1-3.
