# Supplementary material for: On-demand serum-free media formulations for human hematopoietic cell expansion using a high dimensional search algorithm
Source: Commun Biol. 2019 Feb 1;2:48. doi: 10.1038/s42003-019-0296-7 (PMC6358607; doi:10.1038/s42003-019-0296-7)
Supplement: Supplementary file 1 — Supplementary Information [file 42003_2019_296_MOESM1_ESM.pdf]

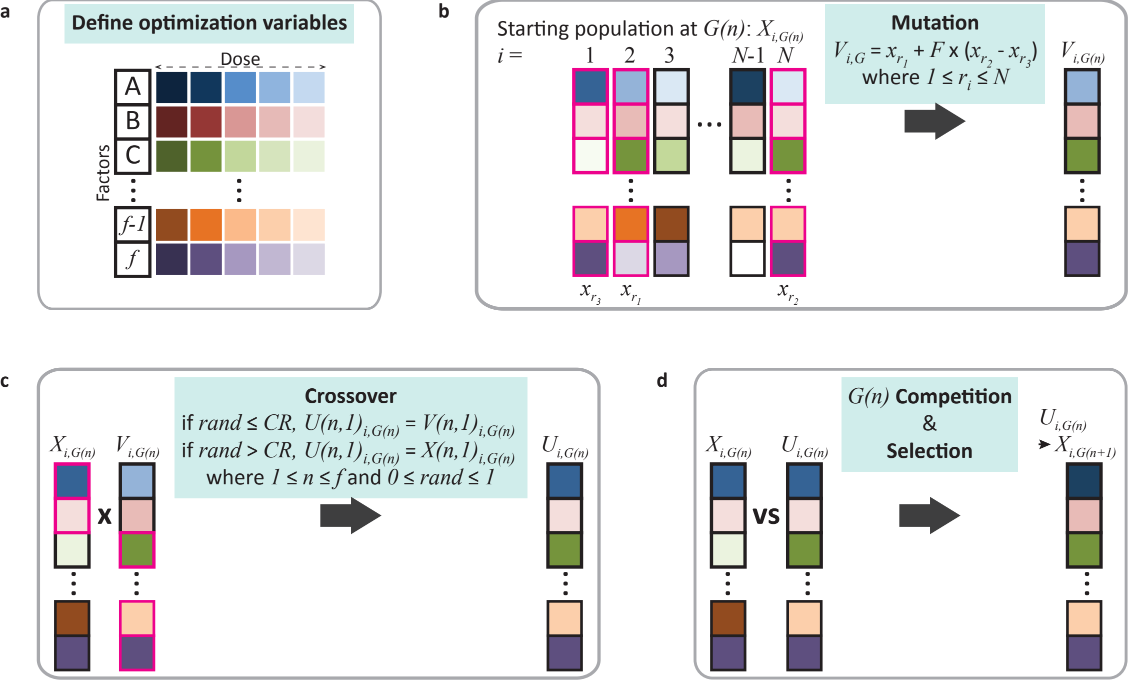

Supplementary Figure 1. A schematic of the classical Differential Evolution (DE) algorithm [19]. Classical DE principles [15] formed the core of the optimization strategy. The DE algorithm works within a defined test population size (N) with randomly generated formulations. The starting set of formulations was designated as the target formulation (X_i, where i = 1 … N) and for each target formulation, a corresponding donor formulation (V_i, where i = 1 … N) was generated through the process of mutation according to the mutation coefficient (F). The donor formulation was further modified by a crossover step according to the crossover constant (CR) producing the trial formulation (U_i, where i = 1 … N) whose score was compared with its corresponding target formulation. The formulation that produced the better score in this competition between the target and trial formulations at each position (i) in the test population set at generation n "$G(n)$" was selected as the target formulation for the next generation at position i (X_i), setting up the algorithm to iterate this process for the next generation.

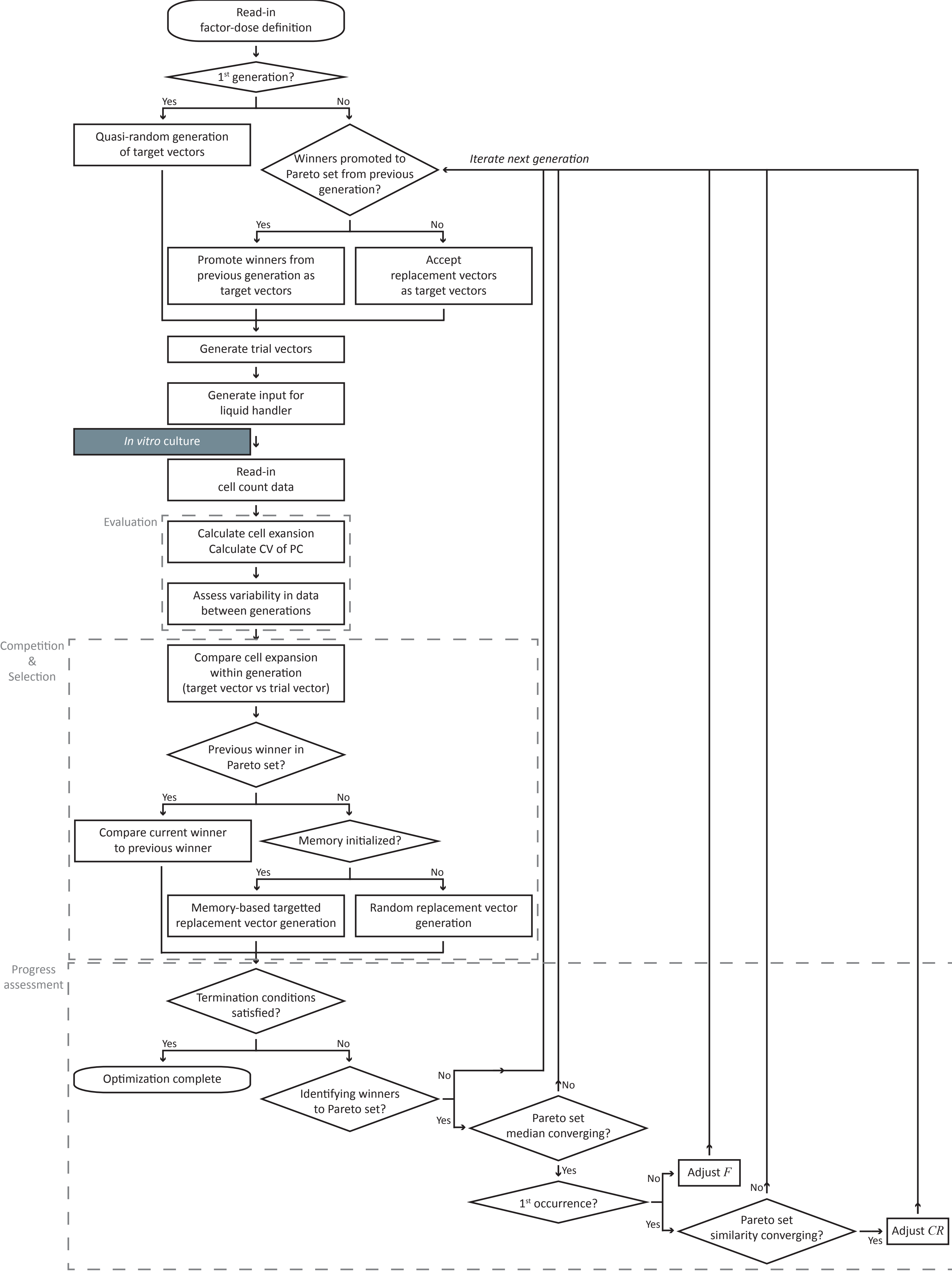

Supplementary Figure 2. Process flow diagram of the HD-DE optimization strategy.

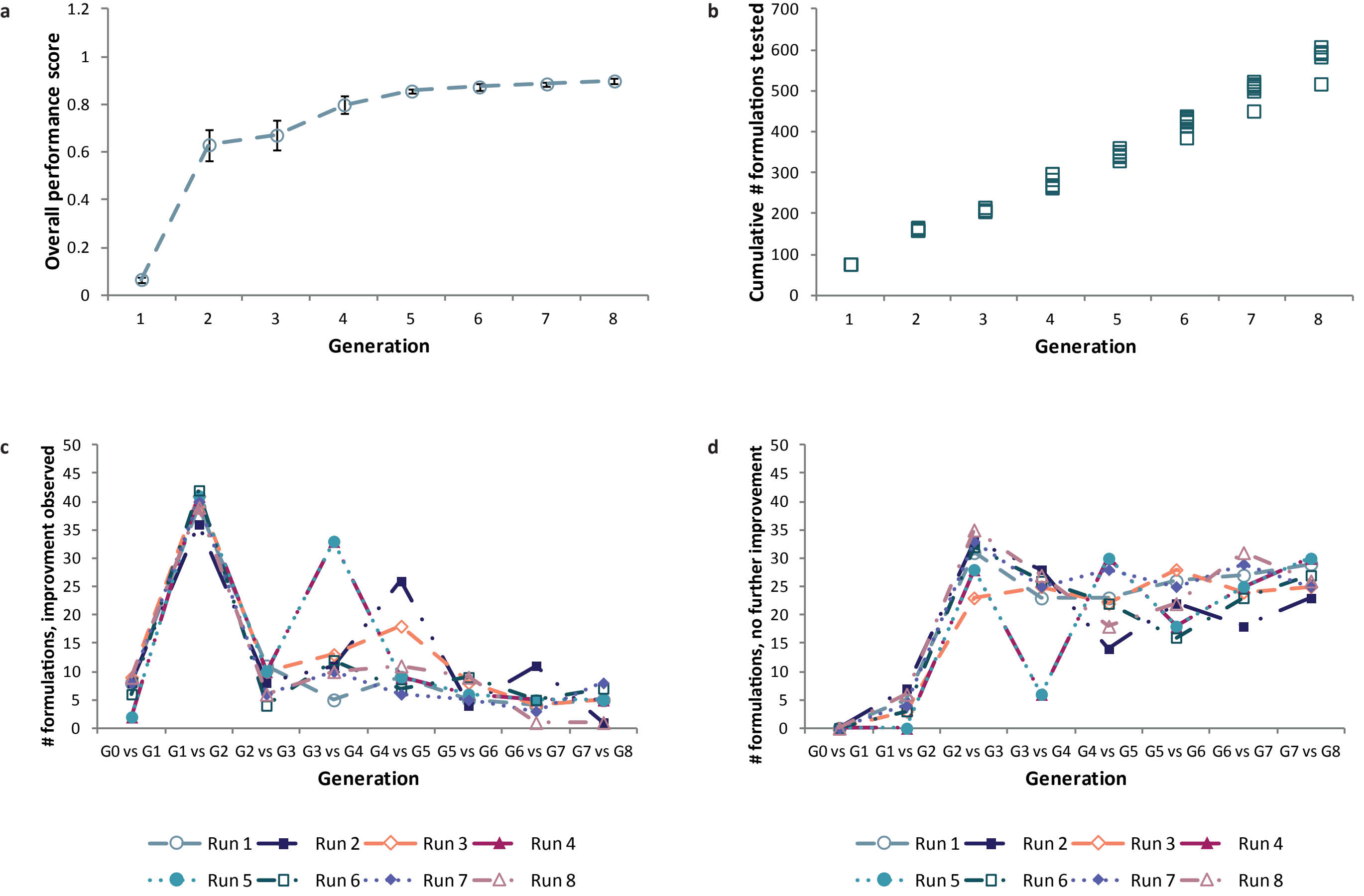

Supplementary Figure 3. Optimization performance of the HD-DE algorithm on a benchmark problem in silico. The Rosenbrock function defined for 15-factors each spanning 5 dose levels was used to generate simulated biological equivalent response in evaluating algorithm performance. (a) The algorithm was able to improve upon the initial conditions to achieve overall improvement and identify optimized conditions. The overall performance was taken as the product of the number of candidate solutions identified as a percentage of the test set size and the average score of the formulations of that generation. The performance was normalized to that of the maximum score possible (theoretical maximum solution of benchmark function). (b) The total number of unique formulations tested tallied at the end of each generation over the entire optimization process. (c) The number of formulations with improved score (i.e. a better combination was identified) decreased towards the end of the optimization. (d) In contrast, the number of formulations without further improvement (previous generation combination carried over to next generation) increased towards the end of the optimization as less number of formulations was replaced by those with improved scores. Data presented 8 independent sets. Data represent mean ± SEM.

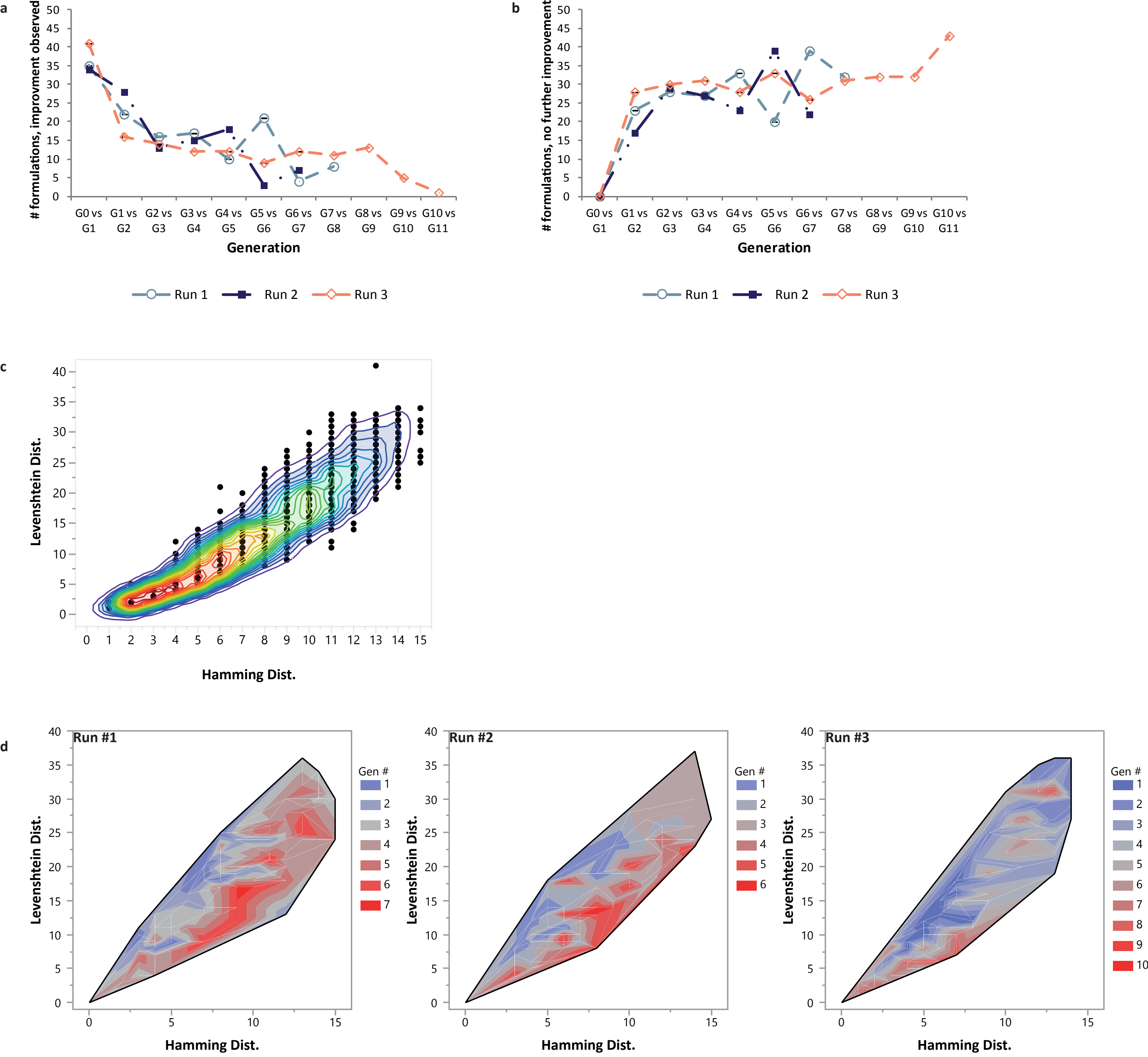

Supplementary Figure 4. In vitro results from 3 independent experimental runs demonstrate robustness of the HD-DE optimization process for TF-1 cells. (a) The number of formulations identified with improved expansion decreased towards the end of the optimization. (b) In contrast, the number of formulations with carried over to the next generation with no further improvement in cell expansion increased towards the end of the optimization. (c) Scatter plot represents a similarity assessment between each formulation of the final candidate solution set with all other formulations. (d) Contour plot distribution of similarity analysis metric coordinates expressed as (Hamming Distance, Levenshtein Distance) between two consecutive generations for each experimental set. Later generations tend to occupy lower half of the contour, suggesting that the formulations undergo smaller changes at later generations.

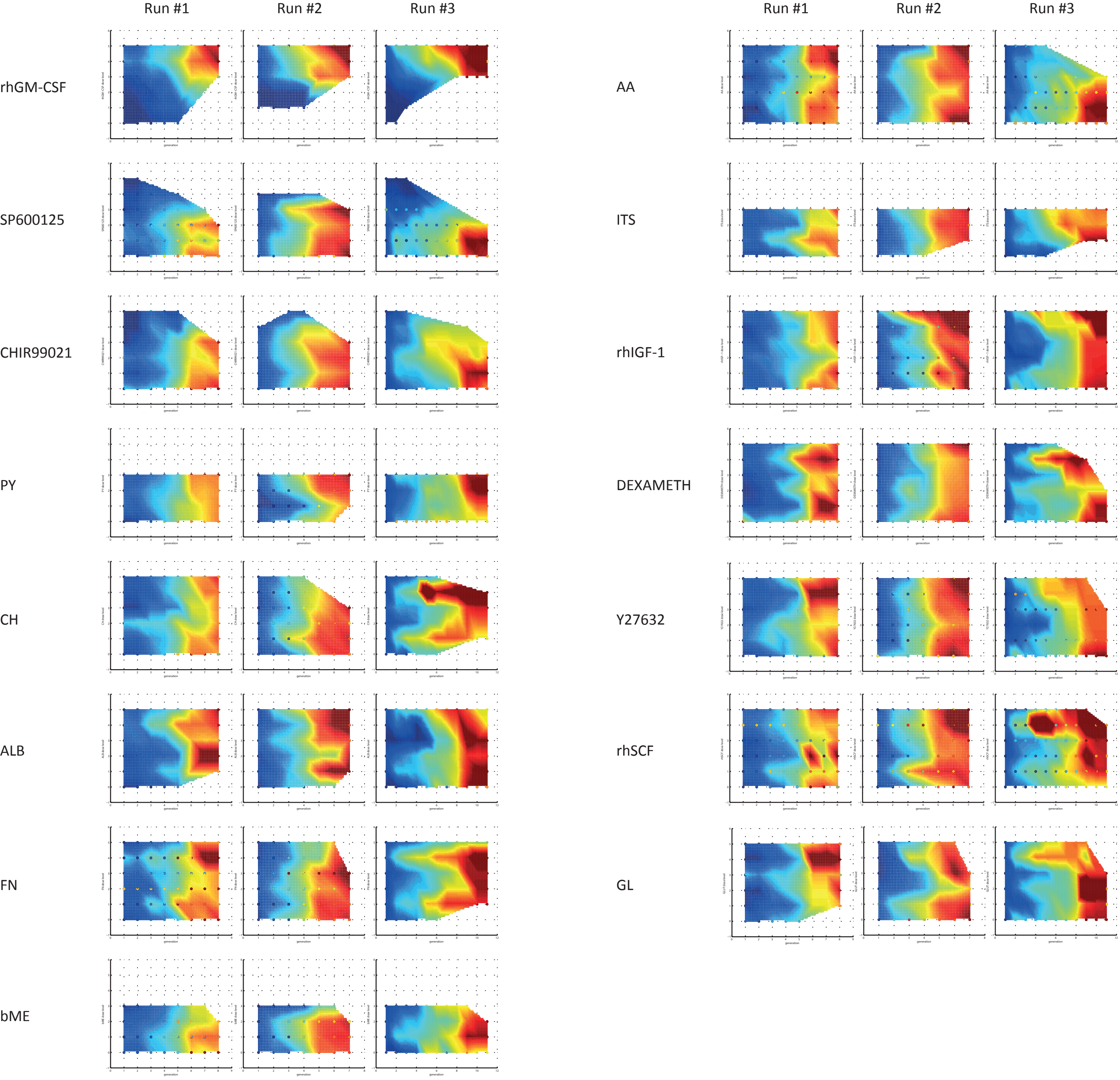

Supplementary Figure 5. Deconstruction of candidate solution set formulations at each generation for TF-1 cells. The change of dose level for each factor (y-axis) over the optimization generations (x-axis) for each candidate formulation of the candidate solution set is plotted. The score at each coordinate (generation, dose level) was averaged and represented according to the average score of the formulations on a heat map colour gradient. The plots illustrate the change in dose levels of single factors within a response generated by a combination of all factors. The most dominant single factor effects show high scoring formulations clustering to high dose level regions for positive effect factors (e.g. rhGM-CSF) and high scoring formulations clustering to low dose level regions for negative effect factors (e.g. SP600125). (See **Supplementary Table 1** for factor legend) .

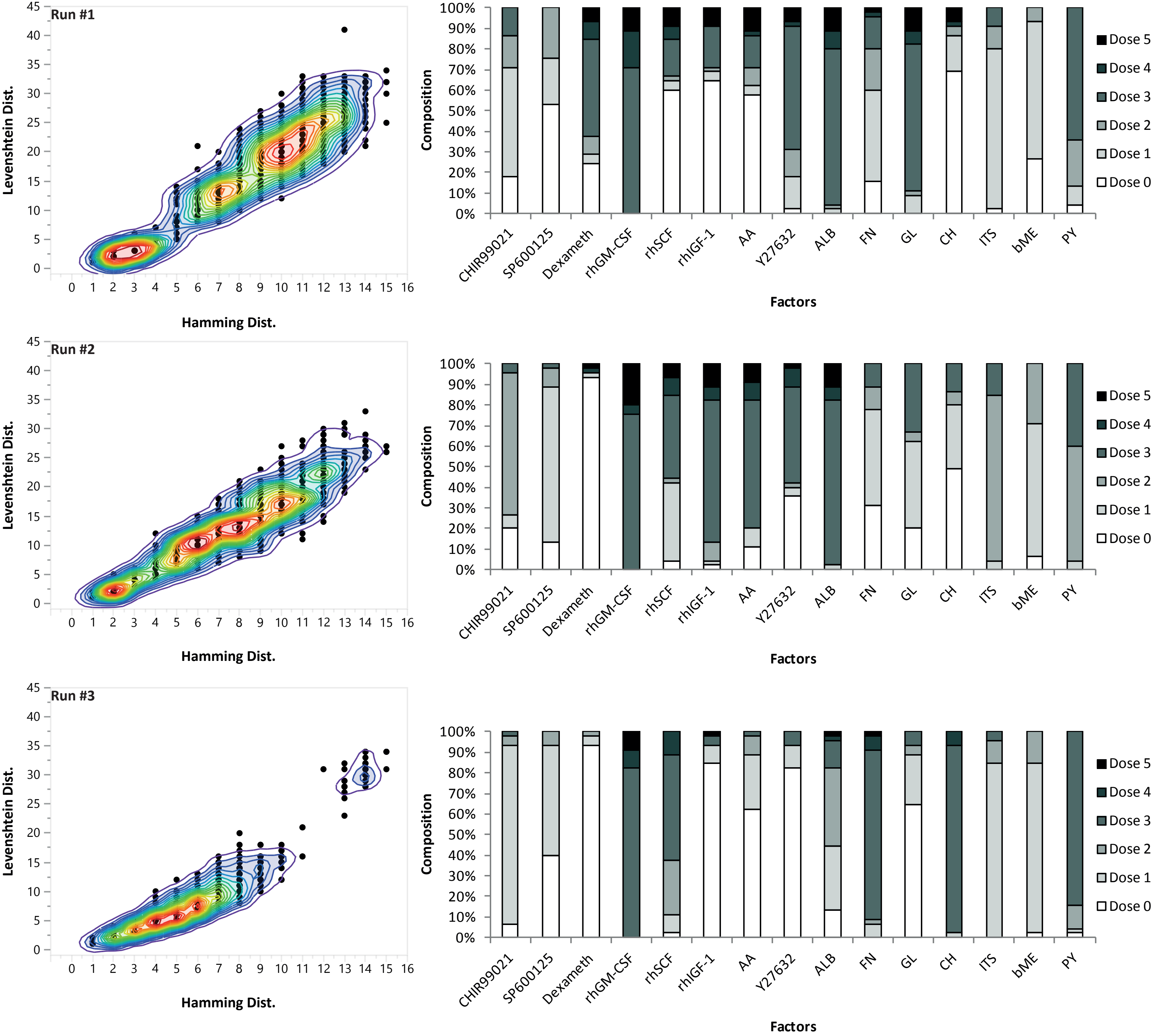

Supplementary Figure 6. Similarity analysis and comparison between individual runs of 15-factor optimization for TF-1 cells. Scatter plot (left panel) with density contour represents a similarity assessment between each formulation of the final candidate solution set (3 experiments separately) with all other formulations. The stacked bar plot (right panel) represents the composition of the final candidate solution set formulations. (See **Supplementary Table 1** for factor legend)

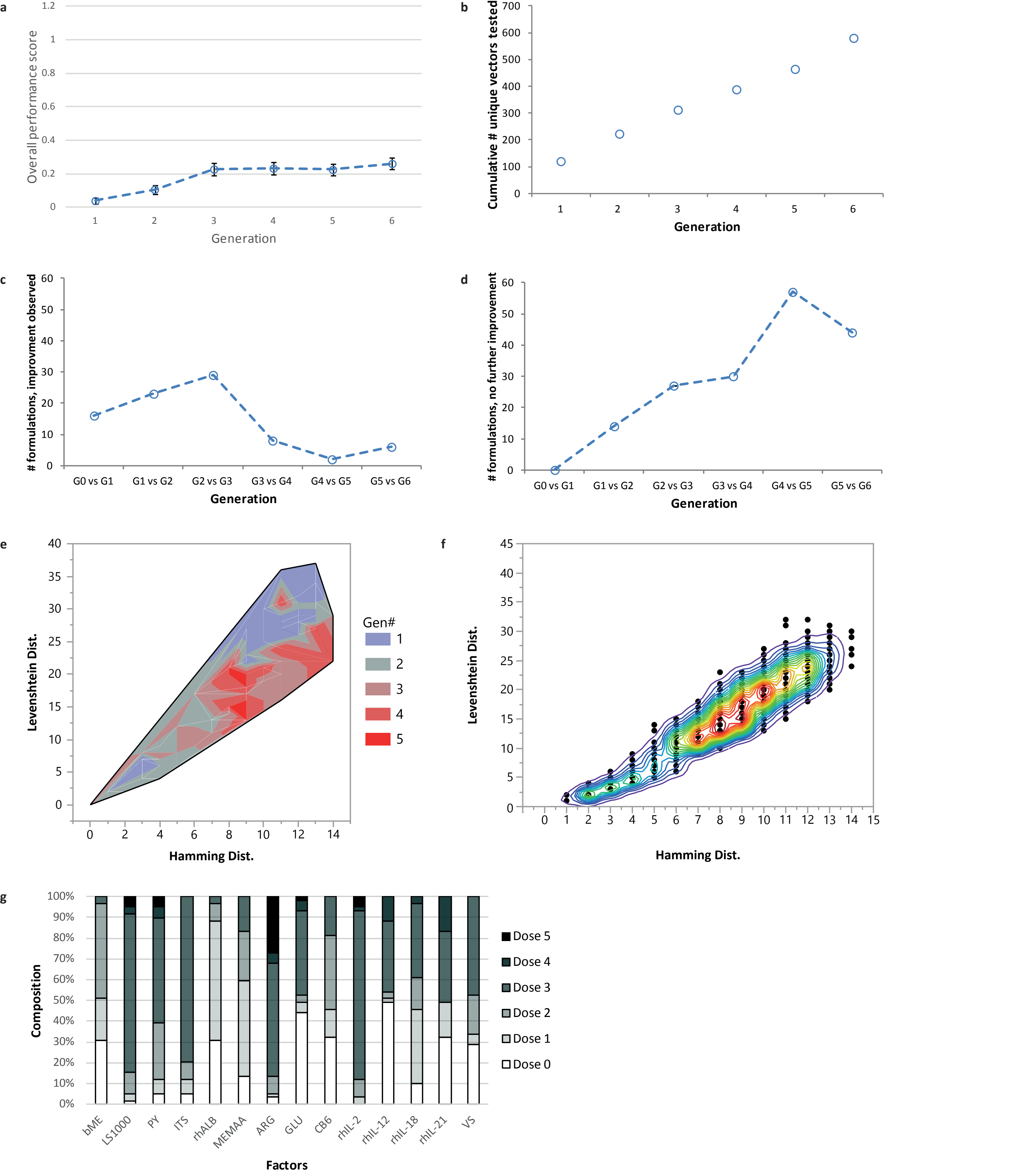

Supplementary Figure 7. Optimization results for 14-factor serum-free T cell expansion culture formulation discovery using HD-DE strategy. (a) The performance was normalized to that of the known maximum score (PC of T cell culture). The overall performance did not reach levels previously observed in TF-1 cell culture. (b) Number of formulations tested increased in a control manner through the generations. (c) The number of formulations with improved score decreased towards the end of the optimization. (d) The number of formulations without further improvement between consecutive generations increased towards the end of the optimization. (e) Scatter plot with density contour representing the similarity assessment between each formulation and all other formulations of the final candidate solution set shows a small cluster of very similar formulations (low Hamming Dist. and low Levenshtein Dist.) while the majority of formulations cluster further away. (f) Contour plot distribution of similarity analysis metric coordinates expressed as (Hamming Distance, Levenshtein Distance) between two consecutive generations of candidate solution sets for each experimental set. Later generations tend to occupy lower half of the contour, suggesting that the formulations undergo smaller changes at later generations. (g) The overall composition of the formulations of the final candidate solution set. Data represent mean ± 95% CI. (See **Supplementary Table 2** for factor legend)

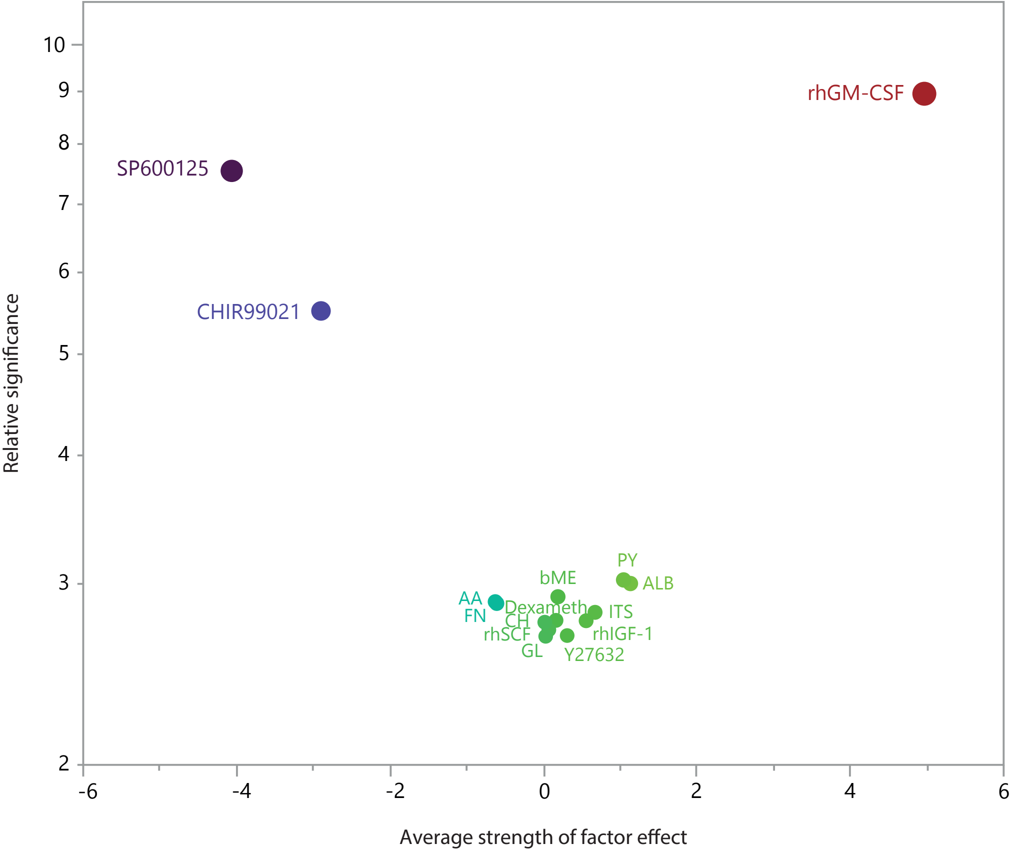

Supplementary Figure 8. Relative significance versus average strength of factor effect of the main factors in TF-1 cell cultures. The average z-score of the FDR-corrected significance and average effect for the single factor effects from each of the 3 independent runs presented in **Figure 2** show the effect characteristics of the factors. (See **Supplementary Table 1** for factor legend)

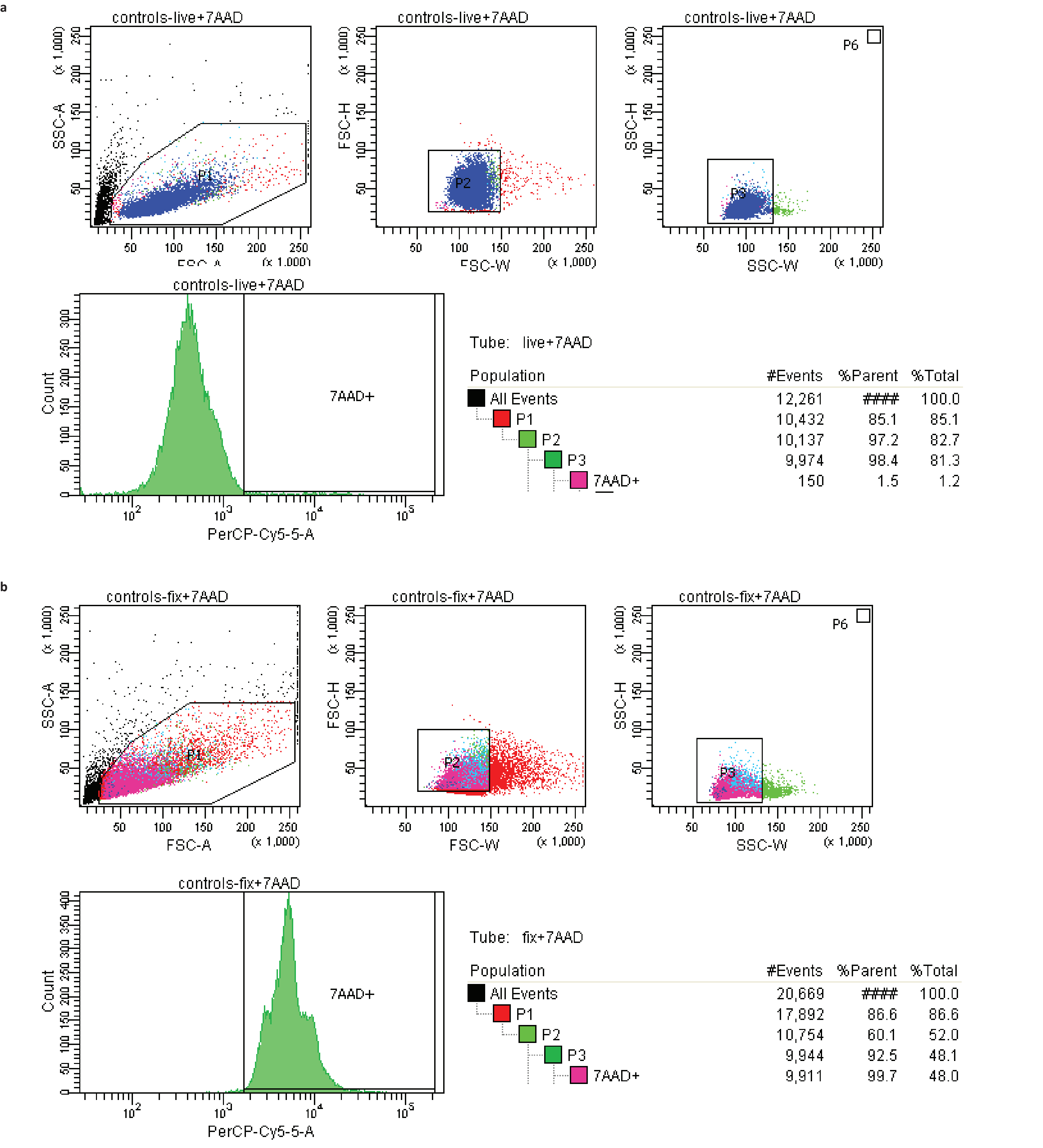

Supplementary Figure 9. Representative flow cytometry plots showing gating strategy for serum-free media optimation using TF-1 cells. (a) The live cell population showed no staining with 7-AAD. (b) The 7-AAD threshold was confirmed with fized cells. (n=3)

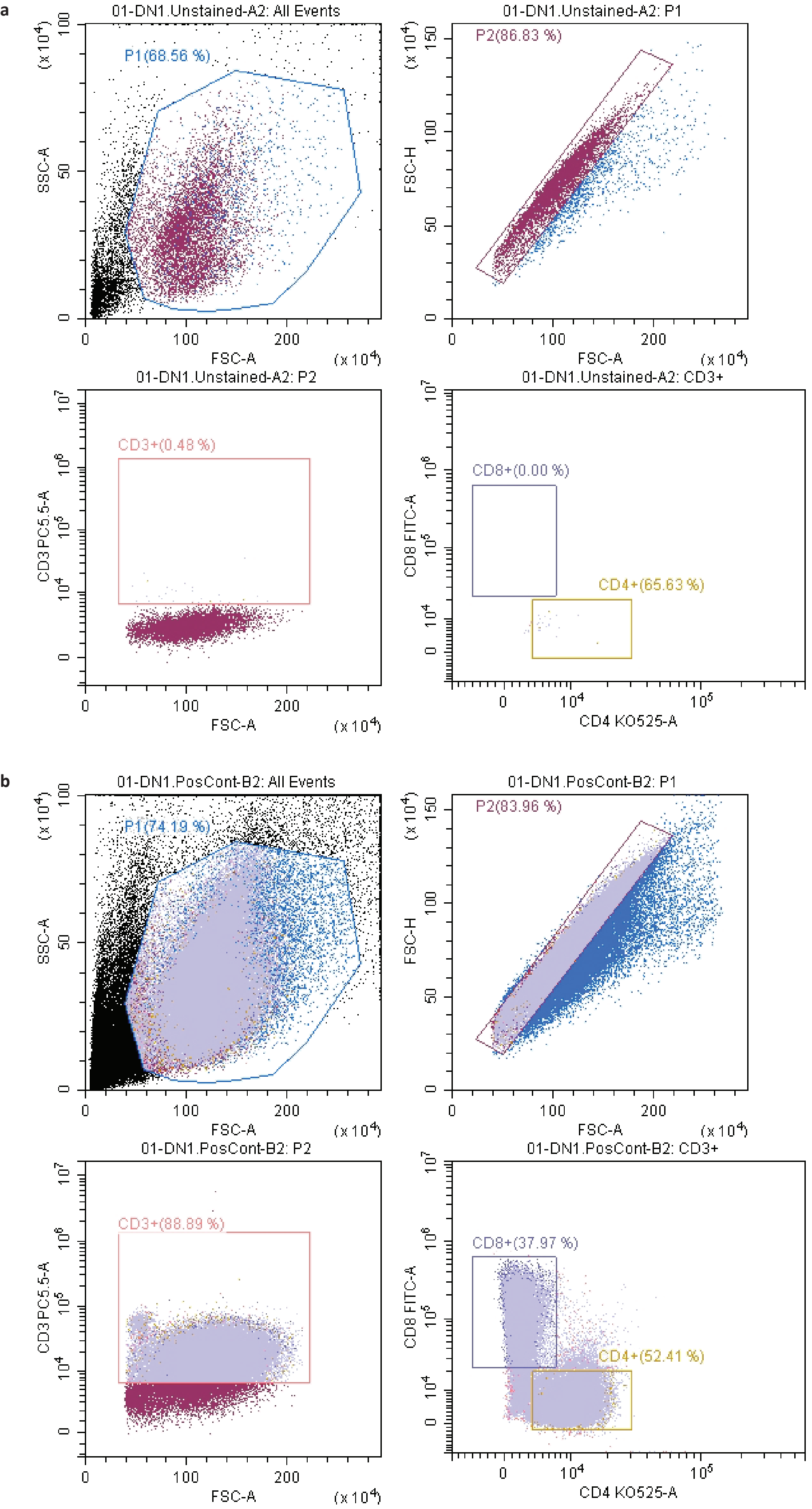

Supplementary Figure 10. Representative flow cytometry plots showing gating strategy for serum-free media optimation using T cells. (a) The unstained cell population showed no staining for CD3, CD4, and CD8 markers. (b) The positive control population cultured under conventional serum-containing conditions showing positive expression of CD3, CD4, and CD8 markers.

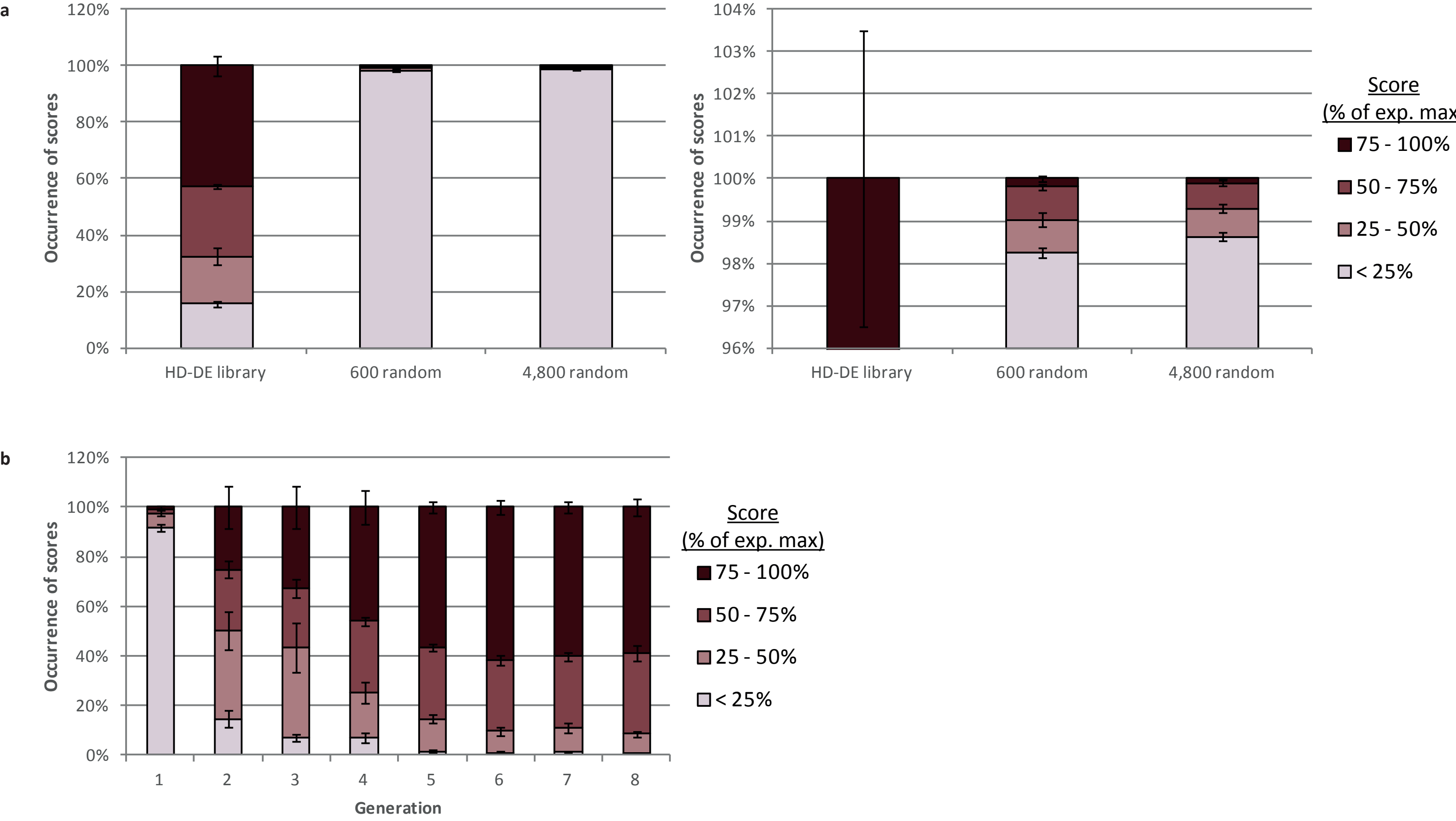

Supplementary Figure 11. Comparison of formulation selection results between HD-DE and random selection. (a) The test library from the 8 runs of HD-DE optimization (**Supplementary Figure 2**) was compared to randomly-generated selection of 600 and 4,800 formulations (6 selections each of 600 or 4,800 formulations). The random formulations were scored on the Rosenbrock function as in **Supplementary Figure 2** and all scores were expressed as % of the theoretical maximum score. Over 8 generations, more than 60% of the formulations encountered by the HD-DE process scored 50% of the max or better, while less than 1% of formulations randomly selected scored at this level. (b) The distribution of scores of the formulations tested from each of the 8 generations of the HD-DE strategy depicts the progression of optimization towards an improved performance state. Data represent mean ± SEM.

Supplementary Tables

Supplementary Table 1. List of 15 factors and corresponding 6 doses that define the scope and scale of in vitro validation of algorithm performance and optimization of serum-free culture condition for TF-1 cell expansion (* Bovine serum albumin (BSA) used as economical substitute of albumin for proof-of-concept and validation experiments in this study).

Factor	Dose levels							Manufac.	Refs
	0	1	2	3	4	5			
Glycogen synthase kinase inhibitor (CHIR99021)	0	0.25	1	3	9.5	30	µM	Reagents Direct	[19, 31]
Jun N-terminal kinase inhibitor (SP600125)	0	0.625	1.875	5	16.625	50	µM	Tocris	[32]
Dexamethasone (Dexameth)	0	1.25	3.125	10	31.875	100	µM	Tocris	[4]
Granulocyte macrophage-colony stimulating factor (rhGM-CSF)	0	0.017	0.067	0.2	0.633	2	ng/ per ml	R&D Systems	
Stem cell factor (rhSCF)	0	0.125	0.375	1	3.125	10	ng/ per ml	R&D Systems	[33]
Insulin-like growth factor 1 (rhIGF-1)	0	0.5	1.5	4	12.5	40	ng/ per ml	R&D Systems	[4]
Ascorbic acid (AA)	0	2.5	6.25	20	63.75	200	µM	Tocris	[4]
Rho kinase inhibitor (Y27632)	0	0.25	0.625	2	6.375	20	µM	Tocris	[19]
Albumin (ALB) *	0	0.025	0.081	0.25	0.788	1.2	mg/ per ml	Sigma-Aldrich	[33]
Fibronectin (FN)	0	0.05	0.15	0.5	1.575	5	µg/ per ml	Sigma-Aldrich	[4]
GlutaMAX™ Supplement (GL)	0	0.02	0.065	0.2	0.63	1	mM	ThermoFisher	[4, 34]
Cholesterol Concentrate (CH)	0	0.01	0.033	0.1	0.315	0.5	%	RMBIO	[4]
ITS Supplement (ITS)	0	0.05	0.158	0.5			x	Sigma-Aldrich	[4, 33]
β-mercaptoethanol (bME)	0	0.025	0.075	0.25			mM	ThermoFisher	[4, 33]
Sodium pyruvate (PY)	0	0.05	0.158	0.5			mM	ThermoFisher	

Supplementary Table 2. List of the 14 factors and corresponding doses that define the scope and scale of algorithm performance and optimization of serum-free culture condition for T cell expansion.

Factor	Dose levels							Manufac.	Refs.
	0	1	2	3	4	5			
β-mercaptoethanol (bME)	0	5.5	17.4	55	173.9	500	μM	ThermoFisher	[35-37]
LS1000 Lipid Supplement (LS1000)	0	0.1	0.3	1	3.2	10	x	GE Healthcare	[38]
Sodium pyruvate (PY)	0	0.05	0.2	0.5	1.6	5	mM	GE Healthcare	[35, 39]
Insulin-Transferrin-Selenium-Ethanolamine (ITS -X)	0	0.1	0.3	1			x	ThermoFisher	[36, 40]
Albumin (rhALB)	0	1	3.2	10			mg/ per ml	Sigma-Aldrich	[36, 38, 41, 42]
MEM Non-Essential Amino Acids Solution (MEMAA)	0	0.1	0.3	1			x	ThermoFisher	[38, 40]
L-Arginine (ARG)	0	0.125	0.4	1.25	4.0	12.5	mM	Sigma-Aldrich	[39]
SG-200 Solution (GLU)	0	0.1	0.3	1	3.2	10	mM	GE Healthcare	[39, 43]
Cell Boost™ 6 (CN-T) Supplement (CB6)	0	0.0003	0.001	0.003			g/ per ml	GE Healthcare	
IL-2 growth factor (rhIL-2)	0	35	55.3	110.7	350	500	IU/ per ml	GE Healthcare	[35, 39, 44]
Interleukin 12 (rhIL-12)	0	1	3.2	10	31.6		ng/ per ml	Stem Cell Technologies	[35, 39]
Recombinant Human IL-18/IL-1F4 Protein, CF (rhIL-18)	0	1	3.2	10	31.6		ng/ per ml	R&D Systems	[35]
Interleukin 21 (rhIL-21)	0	5	7.9	15.8	50		ng/ per ml	Stem Cell Technologies	[45]
MEM Vitamin Solution (VS)	0	0.1	0.3	1			x	ThermoFisher	[38]

Supplementary Table 3. p-values using ANOVA and post hoc Tukey's multiple comparison tests for expression of CD3, CD4, and CD8 for T cells expanded in serum-free media formulations identified through HD-DE optimization (F1-F5) and commercially available media formulation with serum supplementation (PC). n.s. = not significant.

Formulation	-Formulation	CD3+	CD4+	CD8+
PC	F1	n.s.	0.0004	0.0003
PC	F2	n.s.	n.s.	0.0002
PC	F3	n.s.	<.0001	<.0001
PC	F4	n.s.	<.0001	<.0001
PC	F5	n.s.	<.0001	<.0001
F1	F2	<.0001	n.s.	n.s.
F1	F3	0.0283	n.s.	n.s.
F1	F4	n.s.	n.s.	n.s.
F1	F5	0.0444	<.0001	0.0027
F2	F3	n.s.	0.0016	n.s.
F2	F4	0.0015	0.0007	n.s.
F2	F5	n.s.	<.0001	0.0067
F3	F4	n.s.	n.s.	n.s.
F3	F5	n.s.	0.0031	n.s.
F4	F5	n.s.	0.0066	0.0449

Supplementary Table 4. The top 5 serum-free formulations identified using HD-DE for T cell expansion, in dose levels corresponding to the respective tables (as defined in **Supplementary Table 2**).

bME	LS1000	PY	ITS	ALB	MEMAA	ARG	GLU	CB6	IL-2	IL-12	IL-18	IL-21	VS	bME
2	4	3	3	1	1	5	4	3	3	0	3	1	2	2
1	3	3	3	0	2	5	3	0	5	0	1	4	0	1
0	3	3	3	1	1	3	3	0	3	0	0	3	0	0
0	3	3	3	1	2	3	3	0	3	0	1	3	0	0
2	3	0	3	2	1	5	4	2	3	4	4	0	2	2

Supplementary Table 5. The top 5 serum-free formulations identified using HD-DE for TF-1 cell expansion (Run 1-Run 3) in dose levels corresponding to the respective tables (as defined in **Supplementary Table 1**).

Run	CHIR99021	SP600125	Dexameth	rhGM-CSF	rhSCF	rhIGF-1	AA	Y27632	ALB	FN	GLU	CH	ITS	bME	PY
1	1	0	4	4	0	0	0	5	5	1	5	0	1	1	3
1	0	0	4	4	0	0	5	3	5	0	4	5	1	1	3
1	0	2	1	5	0	1	3	1	2	0	4	0	3	1	3
1	0	2	0	4	5	5	4	1	4	4	3	1	1	1	2
1	2	0	5	4	2	3	5	4	5	0	3	1	3	1	2
2	2	1	0	5	5	4	3	3	4	1	1	1	2	2	3
2	0	1	0	5	4	4	0	5	5	3	0	3	2	2	3
2	2	1	0	5	1	3	5	0	3	1	3	0	2	1	2
2	2	1	0	5	5	5	4	4	5	0	1	1	2	1	2
2	2	0	0	5	4	2	3	3	3	0	1	0	2	1	2
3	1	1	0	4	2	0	0	0	2	3	0	3	1	1	3
3	1	1	0	3	3	0	0	0	1	3	2	3	1	1	3
3	1	0	0	3	4	0	0	0	1	3	0	3	1	1	3
3	1	1	0	3	3	0	0	0	0	3	0	4	1	1	3
3	1	0	0	4	2	5	1	0	0	4	3	3	1	1	3

Supplementary Data 1. Comparison of overall performance and number of formulations tested between the Classic DE, Classic DE+var, and HD-DE algorithms. (Excel file available in figshare at https://doi.org/10.6084/m9.figshare.7485665)

Supplementary Data 2. p-values using ANOVA and post hoc Tukey's multiple comparison tests for cell expansion between commercially available media formulations with/without serum supplementation and serum-free formulations identified through HD-DE optimization. (Excel file available in figshare at https://doi.org/10.6084/m9.figshare.7485665)

Supplementary Data 3. Multivariable analysis results using the test formulation library compiled through HD-DE optimization of serum-free media formulations for TF-1 cell expansion (Run 1-Run 3) and T cell expansion listing significance and strength of factor effect. (Excel file available in figshare at https://doi.org/10.6084/m9.figshare.7485665)

Supplementary Discussion

Performance HD-DE strategy against benchmark in silico

For an optimization problem defined for 15-dimensions (factors) with 5 dose levels for each factor, the HD-DE-based optimization strategy demonstrated significant improvement in performance with the integration of components anticipating the unique requirements for analyzing biological response (**Supplementary Fig. 3**). Within 5 generations, the average score over 8 runs of in silico optimization trials have reached 80% of the expected maximum score, continuing to plateau for another 3 generations (**Supplementary Fig. 3a**). On average, this was achieved with the testing of less than 600 unique formulations out of a solution space of over 30 billion possible formulations (**Supplementary Fig. 3b**).

Comparison of the number of formulations with improved score between consecutive generations (**Supplementary Fig. 3c**) with those retained (carried over between consecutive generations) (**Supplementary Fig. 3d**) revealed the convergence of the algorithm towards an optimal state. The number of score improvement occurrence decreased following a sharp spike in the earlier generations while the frequency of carry-over of formulations increased in the later generations. These observations established a baseline behavior profile of the algorithm in the optimization of a problem of similar scale and complexity that could be expected in in vitro optimization.

This optimization result was obtained by searching the equivalent of $2.0x10^{-6}$% of the solution space. Such efficient optimization would be difficult to achieve from the random selection of formulations without the guidance of a selective optimization strategy. The comparison of randomly selected formulations scored on the same benchmark as the in silico runs (**Supplementary Fig. 11a**) demonstrated the optimization ability and efficiency of the HD-DE strategy. At comparable cost (number of formulations tested) to a single run of the HD-DE strategy, 600 formulations were randomly generated. Less than 1% of the formulations scored above 50% of the theoretical maximum score (**Supplementary Fig. 11a**) while HD-DE had encountered more than 60% of all tested formulations to score in the same range (**Supplementary Fig. 11b**). Selection of a larger sample size (4,800 randomly selected, equivalent to the cost of 8 runs of the HD-DE strategy) did not produce any improvement in the scores (**Supplementary Fig. 11a**), demonstrating the difficulty of identifying optimized combinations in a large and complex solution space by random selection.

Composition analysis of 15-factor serum-free formulations for serum-free TF-1 culture

The change in composition of the identified formulations was compared by assessing the degree of similarity between candidate solution sets of consecutive generations. The similarity measurements were represented in (Hamming distance, Levenshtein distance) coordinates and plotted according to sets of consecutive generations. Generally, the formulations were less similar and subject to greater change at earlier generations (greater Hamming distance and/or greater Levenshtein distance measured for same Hamming distance), becoming more similar and subject to smaller changes at later generations (**Supplementary Fig. 4d**). The characteristics of the increased similarity among the formulations of the candidate solution set (**Supplementary Fig. 4d**) resulted from the preferred selection of certain dose levels was reflected in the heatmap plots of the average score of the candidate solution set for each of the (generation, dose level) coordinates for single factors over the dose levels versus generations (**Supplementary Fig. 5**).The plots illustrated the algorithm selectively filtering out ineffective dose regions for factors to cluster the formulations towards high-scoring regions (**Supplementary Fig. 5**). The deconstruction

of single factor effects over the progression of generations and candidate solution set score illustrated the clustering of the positive effect factors and negative effect factors identified through multivariable analysis towards high doses and towards low doses in the high-scoring formulations, respectively. This trend was supported by the clear separation of the single factors with the most significant effects (**Supplementary Fig. 8**). The factors with less significant or insignificant effects (factors represented in green and blue data points in **Supplementary Fig. 8**) also correspond to the heatmap plots of single factor effects (**Supplementary Fig. 5**) that did not reflect a clear selection bias toward either high or low dose level regions. These observations suggest that such factors may not have a significant effect as single factors but contribute toward the overall condition by participating in interactions (**Figure 5b**). The top 5 formulations from each of the experimental runs are identified in **Supplementary Table 5**.
